# Predicting Peri-Operative Cardiorespiratory Adverse Events in Children with Idiopathic Pulmonary Arterial Hypertension Undergoing Cardiac Catheterization Using Echocardiography: A Cohort Study

**DOI:** 10.1007/s00246-024-03447-3

**Published:** 2024-03-21

**Authors:** Timothy J. W. Dawes, Valentine Woodham, Emma Sharkey, Angus McEwan, Graham Derrick, Vivek Muthurangu, Shahin Moledina, Lucy Hepburn

**Affiliations:** 1 Department of Anaesthesia, Great Ormond Street Hospital for Children NHS Foundation Trust, Great Ormond Street, London, WC1N 1LE UK; 2 UCL Institute of Cardiovascular Science, University College London, London, UK; 3 Department of Anaesthesia, Evelina London Children’s Hospital, Guy’s and St. Thomas’ NHS Foundation Trust, London, UK; 4 Department of Paediatric Cardiology, Great Ormond Street Hospital for Children NHS Foundation Trust, London, UK; 5 National Paediatric Pulmonary Hypertension Service UK, Great Ormond Street Hospital for Children NHS Foundation Trust, London, UK

**Keywords:** Pulmonary hypertension, Right ventricle, Anesthesia, Echocardiography, Risk stratification

## Abstract

General anesthesia in children with idiopathic pulmonary arterial hypertension (PAH) carries an increased risk of peri-operative cardiorespiratory complications though risk stratifying individual children pre-operatively remains difficult. We report the incidence and echocardiographic risk factors for adverse events in children with PAH undergoing general anesthesia for cardiac catheterization. Echocardiographic, hemodynamic, and adverse event data from consecutive PAH patients are reported. A multivariable predictive model was developed from echocardiographic variables identified by Bayesian univariable logistic regression. Model performance was reported by area under the curve for receiver operating characteristics (AUC_roc_) and precision/recall (AUC_pr_) and a pre-operative scoring system derived (0–100). Ninety-three children underwent 158 cardiac catheterizations with mean age 8.8 ± 4.6 years. Adverse events (*n* = 42) occurred in 15 patients (16%) during 16 catheterizations (10%) including cardiopulmonary resuscitation (*n* = 5, 3%), electrocardiographic changes (*n* = 3, 2%), significant hypotension (*n* = 2, 1%), stridor (*n* = 1, 1%), and death (*n* = 2, 1%). A multivariable model (age, right ventricular dysfunction, and dilatation, pulmonary and tricuspid regurgitation severity, and maximal velocity) was highly predictive of adverse events (AUC_roc_ 0.86, 95% CI 0.75 to 1.00; AUC_pr_ 0.68, 95% CI 0.50 to 0.91; baseline AUC_pr_ 0.10). Pre-operative risk scores were higher in those who had a subsequent adverse event (median 47, IQR 43 to 53) than in those who did not (median 23, IQR 15 to 33). Pre-operative echocardiography informs the risk of peri-operative adverse events and may therefore be useful both for consent and multi-disciplinary care planning.

**Supplementary Information:**

The online version contains supplementary material available at 10.1007/s00246-024-03447-3.

## Introduction

Pediatric pulmonary hypertension is associated with a five-fold increase in peri-operative cardiovascular morbidity and a seven-fold increase in mortality in children undergoing non-cardiac surgery [1, 2]. Pre-operative risk stratification, multi-disciplinary involvement, and post-operative care planning are recommended [3], though published series include children with a wide variety of cardiac lesions, treatment strategies, and risk profiles [1, 2, 4] even within pulmonary hypertension [5–8]. Pooling data from patients with a broad variety of pathophysiological processes may prevent accurate risk stratification and the identification of relevant physiological disturbances for individual disease processes.

Idiopathic pulmonary arterial hypertension (PAH) is a subgroup of pulmonary hypertension associated with a high incidence of serious peri-operative adverse events [5]. We report the incidence of peri-operative adverse events in children with confirmed PAH undergoing cardiac catheterization and investigate whether features of pre-operative trans-thoracic echocardiography can accurately predict peri-operative adverse events.

## Material and Methods

This is a retrospective cohort study of consecutive incident patients referred to the Great Ormond Street Hospital (GOSH) National Pulmonary Hypertension Service (London, UK). The reporting of this study conforms to the Strengthening the Reporting of Observational Studies in Epidemiology (STROBE) and the Reporting Of Bayes Used in clinical STudies (ROBUST) guidelines, and followed the TRIPOD checklist [9–11]. Study design was sequentially developed with two groups from the GOSH Biomedical Research Centre’s Patient and Participant Involvement department.

### Patient Cohort

Patients referred to the National Paediatric Pulmonary Hypertension Service UK were retrospectively reviewed. Demographic, echocardiographic, anesthetic, clinical, and cardiac catheterization data for patients with a final diagnosis of idiopathic, hereditary, or familial PAH undergoing elective cardiac catheterization under general anesthesia were screened from the patient notes. Procedures undertaken at other centers, without echocardiographic or anesthetic data, or performed under local anesthesia were excluded.

### Echocardiography

Echocardiography was performed to international standards with interpretation by physiologists or cardiologists with advanced echocardiography training. Data were entered on a fixed proforma (Supplementary Table 1). Qualitative measures (mild/moderate/severe) were converted to an ordinal Likert scale (Supplementary Table 2).

### Hemodynamic Monitoring

Lowest intra-operative heart rate, mean arterial blood pressure, and hemoglobin oxygen saturation (SpO_2_) were measured during cardiac catheterization before vasoreactivity testing and expressed as a percentage of values on admission. Hemodynamic measurements at cardiac catheterization were obtained using a balloon-tipped, flow-directed Swan-Ganz catheter (Baxter Healthcare, Irvine, California). Mean pulmonary artery pressure (mPAP) and pulmonary capillary wedge pressure (PCWP) were averaged over the respiratory cycle. After catheterization, the patient was transferred into the magnetic resonance suite with continuing general anesthesia and cardiac output (CO) calculated from phase contrast imaging. Pulmonary vascular resistance (PVR) was calculated as (mPAP–PCWP) / CO.

### Outcome

Adverse events were based on previous published definitions [1] and analyzed as a composite of complications (arrhythmias, cardiac arrest, pulmonary hypertensive crisis, hypotension causing premature termination of the procedure or death, bronchospasm/laryngospasm, and difficult intubation) and unforeseen escalations (initiation of intravenous or inhaled vasoactive therapies, failed extubation/reintubation, post-operative invasive, or non-invasive, respiratory support or emergency admission to intensive care) occurring within 24 h of the induction of anesthesia, since individual adverse events were anticipated to be rare. Adverse events were reported individually, for example, hypotension and electrocardiographic abnormalities were separately reported, even if occurring during the same procedure.

### Statistical Analysis

The incidence of peri-operative adverse events was reported as an unadjusted percentage in relation to the number of catheterization procedures and the number of patients. Adverse events were treated as a composite outcome variable, since the incidence of individual adverse events was anticipated to be low, and statistical modeling therefore unreliable.

Statistical modeling of outcome prediction is described in detail in the Supplementary Statistical Analysis. In outline, the association of pre-operative factors with adverse events was tested by Bayesian univariate mixed-effects logistic regression. A power calculation to inform sample size was performed by simulation using published effect size data [5], and a sensitivity analysis was conducted to demonstrate model stability with different priors. Pre-operative variables associated with adverse events at *p* < 0.05 were used in the multivariable model, divided into ranges representing “low,” “medium,” and “high” risk and added to a multivariable model to predict adverse events using Partial Least Squares regression on a large number (*n* = 1000) of bootstrapped samples. Median coefficients were extracted and rescaled so that patients with “low,” “medium,” or “high” risk characteristics in all variables scored a total of 0, 50, and 100 points, respectively. Inference was calculated by permutation testing. Model performance was assessed by area under the curve for receiver operating characteristics (AUC_roc_) and for precision-recall (AUC_pr_) with 95% credible intervals taken from resampling procedures.

To avoid over-optimistic estimates of model accuracy, an ‘optimism bias’ was calculated, and corrected for, using a bootstrap procedure in line with published guidelines and clinical studies [4, 12, 13]. Optimism bias corrects the reported accuracy of a model which has been developed and tested on the same dataset, providing a more realistic estimate of model performance [13–16]. The scoring system was then applied to the original patient group, scores for each patient were calculated (0–100) and models to predict each outcome (escalation of care, complication and no adverse event) were fitted by logistic regression. The association between pre-operative score and adverse event was confirmed by mixed-effects logistic regression.

### Ethics

The project was registered with the Great Ormond Street Hospital (GOSH) Clinical Audit team and received ethical approval from the Regional Ethics Committee (REF: 17/LO/0008) with individual consent waived as the data were collected during routine clinical care.

## Results

### Study Population Characteristics

**Fig. 1:**
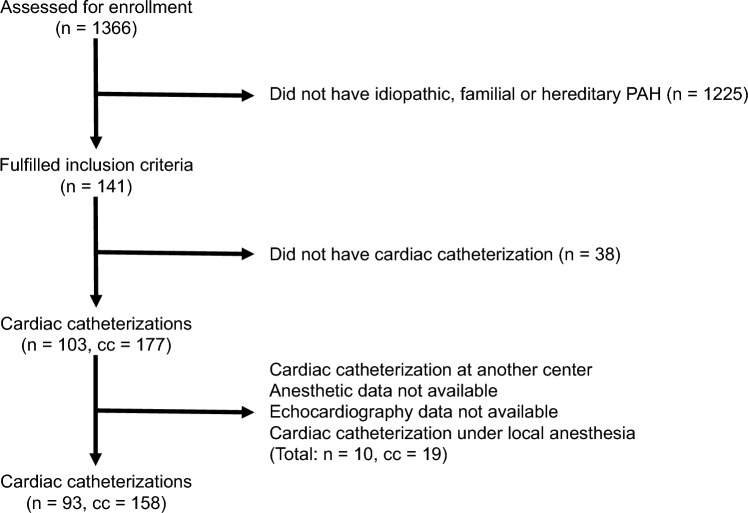
Flow diagram for inclusion of study subjects with number of cardiac catheterizations (cc) and patients (*n*). Exclusion categories with ≤ 3 patients amalgamated with larger groups to avoid potentially identifiable data. *PAH* pulmonary arterial hypertension

In total, 1366 consecutive children referred for investigation to GOSH National Pulmonary Hypertension Service were evaluated for eligibility (Fig. 1) to identify 158 elective cardiac catheterizations (cc) from 93 children (n) under general anesthesia. Children in the final cohort had a mean age of 8.8 ± 4.6 years, weighed 31.6 ± 17.9 kg and were 63% female (Table 1). Heart rate, non-invasive blood pressure and peripheral oxygen saturation (SpO_2_) data were available in all subjects. In 132 procedures (84%), patients had been treated with a pulmonary vasodilator pre-operatively, either a phosphodiesterase 5 inhibitor (cc = 114, 72%), endothelin antagonist (cc = 77, 49%), or a prostacyclin analogue infusion (cc = 41, 26%). Monotherapy, dual therapy, and triple therapy were used before 54 (34%), 56 (35%), and 22 (14%) cardiac catheterizations, respectively.

**Table 1:** Baseline characteristics of patients at the time of cardiac catheterization

		None/Trivial	Mild	Mild/Mod	Mod, Mod/Severe, Severe	n	%
**Demographics**							
Age (years)	8.8 ± 4.6					158	100
Sex (F/M)	99 / 59					158	100
Weight (kg)	31.6 ± 17.9					158	100
Body surface area (m^2^)	1.05 ± 0.40					151	96
**Hemodynamics**							
Mean pulmonary artery pressure (mmHg)	48.2 ± 23.0					155	98
PCWP (mmHg)	8.9 ± 2.7					147	93
Cardiac index (L.min^−1^.m^−2^)	3.0 ± 1.5					133	84
PVRI (WU.m^2^)	15.4 ± 11.8					146	92
**Echocardiography**							
Right ventricular dilatation (%)		9	18	9	114	150	95
Right ventricular hypertrophy (%)		7	20	6	89	122	77
Right ventricular dysfunction (%)		78	33	5	38	154	97
Interventricular septal flattening (Y/N)	90/10					100	63
Fractional area change (%)	28.3 ± 10.6					17	11
TAPSE Z score	-1.9 ± 3.6					102	65
Doppler S’ (cm.s^−1^)	12.3 ± 3.3					50	31
Tricuspid regurgitation severity (%)		63	56	11	27	157	99
Tricuspid regurgitation velocity (m.s^−1^)	4.1 ± 1.0					137	87
Right atrial dilatation (Y/N)	55/10					65	41
Effusion (Y/N)	4/49					53	34
Left ventricular ejection fraction (%)	70 ± 12					38	24
Pulmonary regurgitation velocity (m.s^−1^)	2.8 ± 1.0					45	28
Pulmonary regurgitation end-diastolic velocity (m.s^−1^)	2.5 ± 0.7					67	42
Pulmonary regurgitation severity (%)		73	50	10	4	137	87
Inferior caval dilatation (Y/N)	28/33					61	39
Atrial septal defect (Y/N)	42/81					123	78
Ventricular septal defect (Y/N)	2/68					70	44

Categories with ≤ 3 entries have been amalgamated with larger categories in this table, but not in the analysis, to avoid potentially identifiable data. *F* female, *M* male, *Mod* moderate, *N* no, *PCWP* pulmonary capillary wedge pressure, *PVRI* indexed pulmonary vascular resistance, *TAPSE* tricuspid annular plane systolic excursion, *Y* yes

### Pre-Operative Echocardiography

All patients underwent echocardiography pre-operatively (Supplementary Fig. 1). Right ventricular function was normal (cc = 78, 49%), mildly or mild/moderately impaired (cc = 38, 24%), moderately or moderate/severely impaired (cc = 26, 16%), or severely impaired (cc = 12, 8%) before catheterization. Median tricuspid and pulmonary regurgitation velocities were 4.2 m.s^−1^ (IQR 3.3 to 4.9) and 2.5 m.s^−1^ (IQR 2.0 to 2.8).

### Anesthesia

Patients were anesthetized by a consultant anesthesiologist (cc = 114, 72%), a consultant anesthesiologist, and a senior registrar (cc = 37, 23%) or two consultant anesthesiologists (cc = 7, 4%). No patients had pre-operative respiratory or cardiovascular support. Anxiolytics were administered before 58 procedures (37%) using single-agent midazolam (cc = 43, 27%), temazepam (cc = 13, 8%), or dexmedetomidine (cc = 1, 0.6%). Before one procedure (0.6%), a double premedication of dexmedetomidine and midazolam was administered. Induction of anesthesia was intravenous (cc = 82, 52%) or inhalational (cc = 76, 48%). Intravenous induction was more common in older children (OR 1.1.yr^−1^, 95% CI 1.0 to 1.2). Propofol and fentanyl were the most commonly used induction, and co-induction, agents (cc = 73, 46%; cc = 64, 41%, respectively) though other agents were also used (Supplementary Fig. 3). All patients were positive pressure ventilated via an endotracheal tube (cc = 157, 99%) or a laryngeal mask (cc = 1, 1%).

Patients were maintained on sevoflurane (cc = 70, 44%), isoflurane (cc = 73, 46%), or a propofol infusion (cc = 15, 9%). Remifentanil was infused in 13 catheterizations (cc = 13, 8%), either with propofol (cc = 11, 7%) or with sevoflurane (cc = 2, 1%). Anesthesia led to reductions in mean arterial pressure (median -32%, IQR − 43 to − 22), heart rate (− 18%, IQR − 28 to 0), and SpO_2_ (− 1%, IQR − 4 to + 1). In twelve catheterizations (cc = 12, 8%), one or more vasoactive treatments were administered including phenylephrine (cc = 6, 4%), adrenaline (cc = 2, 1%), isoprenaline (cc = 1, 1%), and inhaled nitric oxide (cc = 6, 4%).

### Cardiac Catheterization

Cardiac catheterization had a median duration of 45 min (IQR 30 to 85) with hemodynamic data acquired in 155 studies (98%) and balloon atrial septostomy performed in 26 studies (cc = 26, 16%). In 3 studies (cc = 3, 2%), hemodynamic data were not acquired due to cardiovascular instability (cc = 1, 1%) or direct progression to atrial septostomy (cc = 2, 1%). Mean pulmonary artery pressure (mPAP) at catheterization had a median value of 44 mmHg (IQR: 30–65) with a median cardiac index (CI) of 2.8 L.min^−1^.m^−2^ (IQR 2.2 to 3.5), which correlated inversely with the degree of right ventricular (RV) dysfunction (*r* = − 0.24, *p* = 0.006). Mean pulmonary artery pressure correlated with echocardiographic measures of pulmonary artery pressure including RV systolic pressure (*r* = 0.61, 95% CI 0.49 to 0.71), tricuspid regurgitation peak velocity (*r* = 0.60, 95% CI 0.48 to 0.70) and pulmonary regurgitation maximal velocity (*r* = 0.47, 95% CI 0.20 to 0.67) (Supplementary Table 3).

Maximal tricuspid regurgitation velocity (4.6 vs 4.0 m.s^−1^, *p* = 0.02) and RV dysfunction (mild/mod vs mild, *p* = 0.03) were greater in patients who underwent atrial septostomy, mean pulmonary artery pressure before septostomy was similar (53 vs 47 mmHg, *p* = 0.31; Supplementary Table 4).

## Adverse Events

**Fig. 2:**
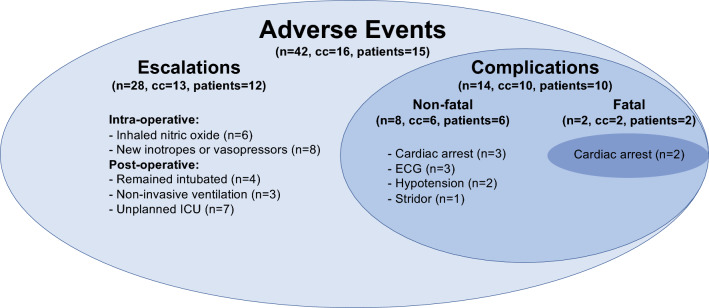
Venn diagram of complications and escalations (collectively: “adverse events”) in the study group. Group summary figures indicate the number of events (*n*), cardiac catheterizations (cc), and patients (patients) affected. *ECG* electrocardiogram, *ICU* intensive care unit

In the peri-operative period, 42 adverse events were recorded in 15 patients (*n* = 15, 16%) during 16 catheterizations (cc = 16, 10%). Twenty eight unexpected escalations of care occurred included the use of intravenous vasoactive therapy (cc = 6, 4%), inhaled vasoactive therapy (cc = 4, 3%), or both (cc = 2, 1%), and post-operative invasive (cc = 4, 3%), or non-invasive (cc = 3, 2%) ventilatory support. Seven procedures (cc = 7, 5%) required unplanned post-operative admission to intensive care. Complications occurred in ten patients (*n* = 10, 11%) during ten catheterizations (cc = 10, 6%) including cardiopulmonary resuscitation (cc = 5, 3%) and death (cc = 2, 1%). Cardiopulmonary resuscitation was initiated after acute desaturation due to pulmonary hypertensive crisis (cc = 3, 2%) and after reductions in cardiac output secondary to bradycardia (cc = 1, 0.6%) and ischemia (cc = 1, 0.6%). The patients who died—one diagnostic and one septostomy procedure—were stable intra-operatively, extubated promptly, and deteriorated during post-operative recovery. Both were in the first half of the series. Electrocardiographic changes were seen in three procedures (cc = 3, 2%). In two procedures, electrocardiographic changes preceded non-fatal cardiac arrests. In a third procedure, they resolved spontaneously without compromise. Two procedures (cc = 2, 1%) were curtailed early because of hypotension. One patient had stridor on extubation which improved without intervention (Fig. 2).

### Outcome Prediction

**Fig. 3:**
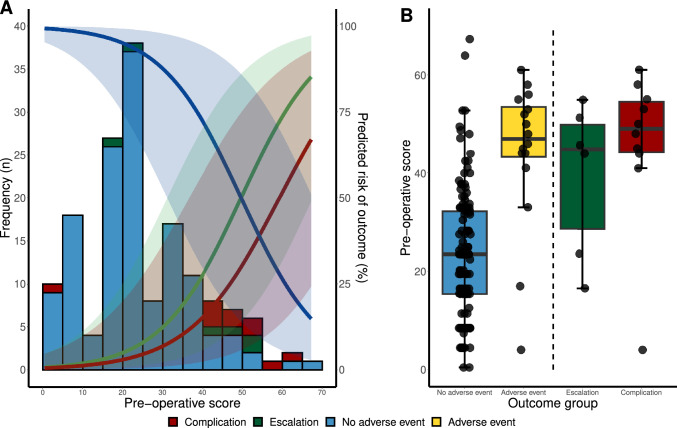
A**.** Histogram of the pre-operative scores (x-axis) for cardiac catheterizations in which a complication (red), escalation (green), or no adverse event (blue) occurred. Pre-operative scores were calculated using the ‘pragmatic’ scoring system. Overlaid curves with shading show the predicted risk (right y-axis) and 95% confidence intervals of these outcomes based on a Bayesian logistic regression model. Predicted risk is capped at the range observed in the data. **B** Boxplot of the pre-operative scores by outcome group. Thick line, box, whiskers, and dots represent the median, inter-quartile range, 1.5 × inter-quartile range, and outliers (> 1.5 × inter-quartile range) in each group, respectively. “Escalation” and “Complication” groups to the right of the dotted line are subgroups of the “Adverse event” group to the left

Younger age, greater RV dysfunction and dilatation, and increased severity and maximal velocity of tricuspid and pulmonary regurgitation were significant univariable predictors of adverse events (Supplementary Table 5) and included in the multivariable model. Pulmonary end-diastolic regurgitant velocity was also a significant univariable predictor, but discarded because of high collinearity with pulmonary regurgitation maximal velocity (*r* = 0.68, *p* = 0.001). The multivariable model predicted adverse events with AUC_roc_ 0.86, (95% CI 0.75 to 1.00; baseline AUC_roc_ 0.50) and AUC_pr_ 0.68 (95% CI 0.50 to 0.91; baseline AUC_pr_ 0.10). An ‘optimized’ scoring system was developed from this model (Supplementary Table 6). To make the model easier to use in clinical practice, risk category boundaries were unified across all the qualitative variables (RV dilatation and function, tricuspid, and pulmonary regurgitation severity) and rounded to whole numbers in the quantitative variables (age, tricuspid, and pulmonary regurgitation velocity). This ‘pragmatic’ scoring system (Table 2) had a prediction performance similar to the optimized scoring system (AUC_roc_ 0.85, 95% CI 0.74 to 1.00; baseline AUC_roc_ 0.50; AUC_pr_ 0.52, 95% CI 0.31 to 0.75; baseline AUC_pr_ 0.10). Using the pragmatic scoring system, pre-operative scores were positively skewed (median 23, IQR 15 to 33; Fig. 3A) and differed significantly between those who had an adverse event (median 47, IQR 43 to 54) and those who did not (median 23, IQR 15 to 32; *z* = 2.1, *p* = 0.04). Pre-operative scores were non-significantly higher in patients who had a complication (median 48, IQR 44 to 54) compared to those who had an escalation but no complication (median 45, IQR 29 to 50, *z* = 0.2, *p* = 0.81). The pre-operative scores of the two patients with fatal complications were 45 and 59.

**Table 2:** Pragmatic scoring system

Measure	Low risk	Points	Medium risk	Points	High risk	Points
Age (years)	> 7.0	− 17	3 – 7	− 13	< 3	− 9
Right ventricular dysfunction	None – Mild	5	Mild/Moderate–Moderate	14	Moderate/Severe – Severe	24
Right ventricular dilatation	None – Mild	3	Mild/Moderate–Moderate	11	Moderate/Severe – Severe	19
Tricuspid regurgitation severity	None – Mild	0	Mild/Moderate–Moderate	5	Moderate/Severe – Severe	9
Tricuspid regurgitation velocity (m.s^−1^)	< 3.0	2	3.0–5.0	9	> 5.0	16
Pulmonary regurgitation severity	None – Mild	7	Mild/Moderate–Moderate	19	Moderate/Severe – Severe	31
Pulmonary regurgitation velocity (m.s^−1^)	< 3.0	0	3.0–5.0	5	> 5.0	10

Points refer to contribution to total risk score (0–100)

There was no association between adverse events and mode of induction (gaseous induction: β = 0.7, 95% CI − 2.0 to 3.5), duration of anesthesia (β = 0.3, 95% CI − 0.9 to 1.6), greatest change in heart rate (β = − 0.58, 95% CI − 1.9 to 0.8), SpO_2_ (β = 0.5, 95% CI − 1.8 to 0.8), or blood pressure (β = − 3.4, 95% CI − 7.8 to 1.0). Neither the number of (0–3) nor the use of (any vs none) pre-operative pulmonary vasodilator treatment was associated with subsequent adverse events (β = 0.4, 95% CI − 1.4 to 2.3; β = 1.6, 95% CI − 7.4 to 10.7, respectively). Procedures which included intra-operative atrial septostomy were associated with a higher incidence of post-operative adverse events (OR 3.8, 95% CI 0.9 to 14.9).

## Discussion

In this retrospective, observational study, we describe the incidence and pre-operative echocardiographic predictors of complications and unexpected escalations of care (collectively: adverse events) in children with idiopathic PAH undergoing cardiac catheterization under general anesthesia. Previous series report the incidence of complications in children with a variety of “severe” congenital heart diseases [1, 2, 4] or across a spectrum of subtypes of pulmonary hypertension [5–8] but to our knowledge, this is the first case series limited to children with idiopathic PAH. Risk stratification data for the survival of children [17, 18] and adults [19, 20] with PH exist, though, to our knowledge, these are the first data describing the peri-operative risks during cardiac catheterization in either group. These data therefore inform discussions about care planning and consent which have been highlighted in recent clinical guidelines, and by the General Medicine Council, as important for clinical practice [3, 21].

The incidence of complications in our series was similar to, or below, that of other published series [5, 6, 8, 22] despite focusing on this high-risk group [5]. Cardiac catheterization in our center is indicated at for new patients at presentation to confirm the diagnosis, or for treated patients to guide treatment in the face of functional or echocardiographic changes. More frequent use of catheterization, during routine follow-up for example, may include more stable patients and be associated with an even lower incidence of adverse events. Conversely, catheterization of patients with severe disease may be associated with a higher incidence of adverse event, as demonstrated by the association in our data between atrial septostomy and adverse peri-operative events. We suggest that children undergoing other non-cardiac procedures, especially longer procedures, or those associated with greater autonomic disturbance and fluid shifts may also be associated with a higher incidence of adverse events [23].

Unlike cardiac catheterization, echocardiography is widely available, well tolerated and can be performed without general anesthesia in children. Despite its technical difficulties in awake children, our data agree with the findings of others that echocardiographic assessments of pulmonary artery pressure [8, 24] and ventricular function [7, 25] have prognostic significance and correlate with invasive measures of afterload [7, 8, 24, 26–28]. The initial, hypertrophic RV response to raised afterload, which serves to lower wall tension and preserve ventricular-arterial coupling [29], was not prognostically significant in our setting. Instead, the progression to RV dilatation – an adaptation which maintains resting stroke volume – and onward to RV dysfunction was associated with a higher risk of subsequent adverse events. It is unclear whether this excess risk is precipitated by an increase in PVR (due to positive pressure ventilation, hypoxia, hypercarbia, fluid shifts, or sympathetic stimulation), or a reduction in coronary blood flow, venous return, or myocardial contractility [30, 31]. However, this later stage of physiological deterioration has repeatedly been associated with poor long-term outcome, poor response to treatment, and now an elevated risk of peri-operative adverse events [32–34].

Our series does not demonstrate a protective role for pre-operative pulmonary vasodilator therapy on the incidence of adverse events, though the timing, dose and class of treatment used pre-operatively was not standardized and our study was not powered to address this question. We note that other guidelines recommend the continuation, modification to intravenous or inhaled equivalents, or escalation of therapy in the peri-operative period [30, 35], and cessation of PH medications can lead to rebound PH and adverse outcomes [36].

Understanding the progression of RV dysfunction and its implications for patient care is an important goal and magnetic resonance imaging remains the gold-standard modality for phenotyping the right ventricle in pulmonary hypertension [37]. Combining image phenotypes with contemporary image acquisition and processing has the potential to improve mechanistic understanding of RV dysfunction, risk stratification, and identify patients suitable for earlier treatment with improvements in long-term outcomes and peri-operative risk [33, 38–41].

## Limitations

These findings are from children with PAH at a single center and cannot be extrapolated to other causes of pulmonary hypertension or children with PAH undergoing other procedures. External validation is paramount for confirmation. History, examination, functional class, and serum blood tests such as NT-proBNP findings are used in other guidelines [17, 18] and may provide exercise-induced or biochemical evidence of cardiac dysfunction not detected by resting echocardiography, though these data were not available for all subjects in our series. Invasive hemodynamic measurements and cardiac output were obtained in the catheterization laboratory and the magnetic resonance suite sequentially, and it is possible that synchronous measurements would provide different derived hemodynamic estimates and insights. The definitions of adverse events were based on definitions in previous similar work [1] though guidelines [42] and clinical practice [43] vary, which may alter group membership and predictive modeling.

We note that our group includes patients with serious adverse events who had low pre-operative risk scores and patients with high pre-operative risk scores who did not have an adverse event. Indications for catheterization, protocols for echocardiography and anesthetic technique, and screening for adverse events were not protocolized and it is possible that the choices made for individual patients biased the recruitment, assessment, peri-operative cardiorespiratory risk, and reporting of adverse outcomes, respectively. Other peri-operative data not reported here, and other assessments which are difficult to quantify, may be important when assessing patient risk. Prospective recruitment and external validation may improve our understanding of the most complete set of predictors and outcomes and the most robust prediction. Lastly, patients’ views on the relative importance of particular adverse events may vary and consent discussions should explore patients’ needs, values, and priorities as recommended by GMC guidance [21].

## Conclusion

Safe anesthesia for children with PAH undergoing cardiac catheterization requires a multi-disciplinary approach. Pre-operative echocardiography may be valuable in informing the consent and care planning processes for these children.

## Supplementary Information

Below is the link to the electronic supplementary material.

Supplementary file1 (DOCX 983 kb)
