# Supplementary material for: Predicting Peri-Operative Cardiorespiratory Adverse Events in Children with Idiopathic Pulmonary Arterial Hypertension Undergoing Cardiac Catheterization Using Echocardiography: A Cohort Study
Source: Pediatr Cardiol. 2024 Mar 21;46(2):475–84. doi: 10.1007/s00246-024-03447-3 (PMC11787273; doi:10.1007/s00246-024-03447-3)
Supplement: Supplementary file 1 — Supplementary file1 (DOCX 983 kb) [file 246_2024_3447_MOESM1_ESM.docx]

## Supplementary Tables

Supplementary Table 1

|  | Quantitative | Qualitative |
| --- | --- | --- |
| Timing | Date  Interval before cardiac catheterization (days) |  |
| Right heart | RV fractional area change  TAPSE Z score  Doppler S’ | Right atrial dilatation  RV dilatation  RV hypertrophy  RV dysfunction  Interventricular septal flattening  Pulmonary arterial dilatation  Pericardial effusion |
| Left heart | LV ejection fraction |  |
| Trans-valvar flow | Tricuspid regurgitation maximal velocity  Pulmonary regurgitation maximal velocity | Tricuspid regurgitation severity  Pulmonary regurgitation severity  Inferior vena caval dilatation |
| Septal |  | ASD  VSD |
| ASD = atrial septal defect; CI = credible interval; LV = left ventricular; mPAP = mean pulmonary artery pressure; PR = pulmonary regurgitation; RV = right ventricular; RVSP = right ventricular systolic pressure; TAPSE = tricuspid annular plane systolic excursion; TR = tricuspid regurgitation; VSD = ventricular septal defect. | | |

| Supplementary Table 2 |  |  |
| --- | --- | --- |
| **Likert scale** | **Valvular regurgitation** | **Ventricular dysfunction** |
| 1 | None | Normal function, preserved function |
| 2 | Trivial, trace, whiff | Reasonable function |
| 3 | Mild | Mild dysfunction |
| 4 | Mild+, mild/moderate | Mild/moderate dysfunction |
| 5 | Moderate, no descriptor | Moderate dysfunction |
| 6 | Moderate+, moderate/severe | Moderate/severe dysfunction |
| 7 | Severe, very, ++ | Severe dysfunction, hypocontractile, gross dysfunction |
| Qualitative terms identified on review of echocardiography reports used to describe the degree of valvular regurgitation or ventricular dysfunction with mapping to the Likert scale. | | |

### Supplementary Table 3

| **Parameter** | **mPAP** | | **95% Credible Intervals** | | **P-value** |
| --- | --- | --- | --- | --- | --- |
|  | **Correlation coefficient** | **Mean difference** | **Lower** | **Upper** |  |
| ASD |  | 3.5 | -5.3 | 12.3 | 0.43 |
| Effusion |  | 35.4 | -29.3 | 100.1 | 0.18 |
| PA dilatation |  | -10.5 | -33.9 | 12.9 | 0.31 |
| PR severity | 0.17 |  | 0.003 | 0.33 | 0.05 |
| PR Vmax | 0.47 |  | 0.20 | 0.67 | 0.001 |
| RA dilatation |  | -6.8 | -25.2 | 11.6 | 0.44 |
| RV dilatation | 0.37 |  | 0.22 | 0.50 | < 0.001 |
| RV dysfunction | 0.33 |  | 0.18 | 0.46 | < 0.001 |
| RV hypertrophy | 0.33 |  | 0.16 | 0.48 | < 0.001 |
| RV systolic pressure | 0.61 |  | 0.49 | 0.71 | < 0.001 |
| Septal flattening |  | -1.6 | -16.9 | 13.7 | 0.82 |
| TAPSE | -0.22 |  | -0.40 | -0.03 | 0.03 |
| TR severity | 0.38 |  | 0.24 | 0.51 | < 0.001 |
| TR velocity | 0.60 |  | 0.48 | 0.70 | < 0.001 |
| Age | -0.07 |  | -0.22 | 0.09 | 0.42 |
| Association of invasive mean pulmonary artery pressure (mPAP) with echocardiographic variables measured by Pearson’s correlation coefficient (continuous and ordinal variables) or unpaired t-test (categorical variables). ASD = atrial septal defect; CI = credible interval; mPAP = mean pulmonary artery pressure; PR = pulmonary regurgitation; RA = right atrial; RV = right ventricular; TAPSE = tricuspid annular plane systolic excursion; TR = tricuspid regurgitation. | | | | | |

### Supplementary Table 4

|  | | **Underwent septostomy** | | | | | **Did not undergo septostomy** | | | | |  |  |
| --- | --- | --- | --- | --- | --- | --- | --- | --- | --- | --- | --- | --- | --- |
|  | |  | **None/Trivial** | **Mild** | **Mild/Mod** | **Mod, Mod/Severe, Severe** |  | **None/Trivial** | **Mild** | **Mild/Mod** | **Mod, Mod/Severe, Severe** | **n** | **%** |
| **Demographics** | |  |  |  |  |  |  |  |  |  |  |  |  |
|  | Age (years) | 7.8 ± 5.1 |  |  |  |  | 9.0 ± 4.5 |  |  |  |  | 158 | 100 |
|  | Sex (F/M) | 18 / 8 |  |  |  |  | 81 / 51 |  |  |  |  | 158 | 100 |
|  | Weight (kg) | 28.6 ± 19.6 |  |  |  |  | 32.2 ± 17.6 |  |  |  |  | 158 | 100 |
|  | Body surface area (m^2^) | 1.01 ± 0.43 |  |  |  |  | 1.06 ± 0.39 |  |  |  |  | 151 | 96 |
| **Hemodynamics** | |  |  |  |  |  |  |  |  |  |  |  |  |
|  | Mean pulmonary artery pressure (mmHg) | 52.6 ± 29.3 |  |  |  |  | 47.4 ± 21.6 |  |  |  |  | 155 | 98 |
|  | PCWP (mmHg) | 7.9 ± 3.4 |  |  |  |  | 9.1 ± 2.6 |  |  |  |  | 147 | 93 |
|  | Cardiac index (L.min^-1^.m^-2^) | 2.3 ± 0.9 |  |  |  |  | 3.1 ± 1.5 |  |  |  |  | 133 | 84 |
|  | PVRI (WU.m^2^) | 21.0 ± 15.3 |  |  |  |  | 14.4 ± 10.9 |  |  |  |  | 146 | 92 |
| **Echocardiography** | |  |  |  |  |  |  |  |  |  |  |  |  |
|  | Right ventricular dilatation (%) |  | 12 | 8 | 62 | 19 |  | 19 | 6 | 60 | 15 | 150 | 95 |
|  | Right ventricular hypertrophy (%) |  | 17 | 0 | 67 | 17 |  | 23 | 6 | 63 | 8 | 122 | 77 |
|  | Right ventricular dysfunction (%) |  | 58 | 8 | 25 | 8 |  | 75 | 2 | 15 | 8 | 154 | 97 |
|  | Interventricular septal flattening (Y/N) | 14/2 |  |  |  |  | 76/8 |  |  |  |  | 100 | 63 |
|  | Fractional area change (%) | 19.0 ± 0 |  |  |  |  | 30.3 ± 10.7 |  |  |  |  | 17 | 11 |
|  | TAPSE Z score | -2.4 ± 3.3 |  |  |  |  | -1.9 ± 3.6 |  |  |  |  | 102 | 65 |
|  | Doppler S’ (cm.s^-1^) | 13.0 ± 5.7 |  |  |  |  | 12.3 ± 3.3 |  |  |  |  | 50 | 31 |
|  | TR severity (%) |  | 62 | 4 | 15 | 19 |  | 79 | 8 | 11 | 2 | 157 | 99 |
|  | TR velocity (m.s^-1^) | 4.6 ± 1.0 |  |  |  |  | 4.0 ± 1.0 |  |  |  |  | 137 | 87 |
|  | Right atrial dilatation (Y/N) | 8/0 |  |  |  |  | 47/10 |  |  |  |  | 65 | 41 |
|  | Effusion (Y/N) | 11/15 |  |  |  |  | 42/90 |  |  |  |  | 53 | 34 |
|  | Left ventricular ejection fraction (%) | 59 ± 11 |  |  |  |  | 71 ± 11 |  |  |  |  | 38 | 24 |
|  | PR velocity (m.s^-1^) | 3.3 ± 1.2 |  |  |  |  | 2.6 ± 0.9 |  |  |  |  | 45 | 28 |
|  | PR end-diastolic velocity (m.s^-1^) | 2.6 ± 0.2 |  |  |  |  | 2.4 ± 0.8 |  |  |  |  | 67 | 42 |
|  | PR severity (%) |  | 82 | 14 | 5 | 0 |  | 91 | 6 | 2 | 1 | 137 | 87 |
|  | Inferior caval dilatation (Y/N) | 7/0 |  |  |  |  |  | 21/33 |  |  |  | 61 | 39 |
|  | Atrial septal defect (Y/N) | 6/15 |  |  |  |  |  | 36/66 |  |  |  | 123 | 78 |
|  | Ventricular septal defect (Y/N) | 1/9 |  |  |  |  |  | 1/59 |  |  |  | 70 | 44 |

F = female; M = male; Mod = moderate; N = no; PCWP = pulmonary capillary wedge pressure; PR = pulmonary regurgitation; PVRI = indexed pulmonary vascular resistance; TAPSE = tricuspid annular plane systolic excursion; TR = tricuspid regurgitation; Y = yes.

### Supplementary Table 5

| **Variable** | **Mean Posterior** | **95% Credible Interval** | | **P-Value** |
| --- | --- | --- | --- | --- |
|  |  | **Lower** | **Upper** |  |
| Age | -0.01 | -0.02 | < -0.001 | 0.04 |
| Atrial septal defect | -0.08 | -0.19 | 0.04 | 0.19 |
| LV fractional shortening | 0.003 | -0.006 | 0.01 | 0.55 |
| Pericardial effusion | 0.02 | -0.34 | 0.38 | 0.96 |
| Pulmonary artery dilatation | 0.08 | -0.08 | 0.25 | 0.35 |
| PR end-diastolic regurgitant velocity | 0.05 | 0.01 | 0.10 | 0.01 |
| PR severity | 0.07 | 0.02 | 0.12 | 0.001 |
| PR velocity | 0.07 | 0.03 | 0.12 | < 0.001 |
| RA dilatation | 0.01 | -0.12 | 0.13 | 0.86 |
| RV dilatation | 0.05 | 0.02 | 0.08 | < 0.001 |
| RV dysfunction | 0.07 | 0.05 | 0.09 | < 0.001 |
| RV fractional area change | < 0.001 | -0.001 | 0.001 | 0.89 |
| RV hypertrophy | 0.02 | -0.02 | 0.06 | 0.39 |
| Interventricular septal flattening | -0.02 | -0.18 | 0.13 | 0.75 |
| TR severity | 0.04 | 0.003 | 0.08 | 0.03 |
| TR velocity | 0.08 | 0.04 | 0.12 | < 0.001 |
| Ventricular septal defect | -0.12 | -0.46 | 0.25 | 0.49 |

Coefficients from the posterior distribution generated from univariable Bayesian logistic regression. Dependent variable was the occurrence of an adverse event, independent variables as listed above. LV = left ventricular; PR = pulmonary regurgitation; RA = right atrial; RV = right ventricular; TR = tricuspid regurgitation.

| Supplementary Table 5 |  |  |  |  |  |  |
| --- | --- | --- | --- | --- | --- | --- |
| **Measure** | **Low Risk** | **Points** | **Medium Risk** | **Points** | **High Risk** | **Points** |
| Age (years) | > 8.0 | -20 | 3.8 – 8.0 | -15 | < 3.8 | -9 |
| RV dysfunction | None – Mild/Moderate | 6 | Moderate – Moderate/Severe | 16 | Severe | 26 |
| RV dilatation | None – Mild/Moderate | 5 | Moderate – Moderate/Severe | 14 | Severe | 22 |
| TR severity | None – Mild/Moderate | 2 | Moderate | 9 | Moderate/Severe – Severe | 16 |
| TR velocity (m/s) | < 4.0 | 4 | 4.0 – 5.0 | 12 | > 5.0 | 20 |
| PR severity | None – Trivial | 3 | Mild – Mild/Moderate | 10 | Moderate – Severe | 17 |
| PR velocity (m/s) | < 3.4 | 0 | 3.4 – 4.4 | 4 | > 4.4 | 8 |
| Percent risk (95% confidence intervals) are for adverse event or complication if all the variables fall in the “low”, “medium” or “high” risk categories. PR = pulmonary regurgitation; RV = right ventricular; TR = tricuspid regurgitation. | | | | | | |

## Supplementary Figures

| Supplementary Figure 1 |
| --- |
|  |
| Summary of statistical methods. Echocardiography variables were divided into categories using k-means clustering and selected for a multivariable model based on univariate association with adverse events (p<0.05). A multivariable partial least squares (PLS) model was built and tested on the full sample and an ‘optimism bias’ subtracted to provide a more realistic estimate of model performance. From a bootstrap sample, a model was fitted and then tested on the full sample and the bootstrap sample with the difference in performance described as the optimism bias. This process was repeated for 1,000 bootstrap samples and then mean optimism bias subtracted from the original performance. Model accuracy was estimated as this ‘optimism bias’ corrected estimate of performance. AUC=Area Under the Curve for Receiver Operating Characteristics (AUC_roc_) or Precision-Recall (AUC_pr_), PLS=Partial Least Squares. |

| Supplementary Figure 2 |
| --- |
|  |
| Pre-operative echocardiographic parameters for the study population. (A) Scatterplot showing the qualitative descriptions of right ventricular (RV) morphology, tricuspid regurgitation (TR) and pulmonary regurgitation (PR) severity. All patient assessments were converted to a seven-point Likert scale (y-axis). (B) Boxplot with overlying scatterplot showing the quantitative TR and PR maximal velocities. Thick line, box, whiskers, and dots represent the median, inter-quartile range (IQR), 1.5 x IQR, and outliers (>1.5 x IQR) in each group, respectively. |

### Supplementary Figure 3

| **** |
| --- |
| Grouped barchart showing the frequency with which each pharmacological agent was used at induction, as a percentage of all cases. IV = intravenous. |

| Supplementary Figure 4 |
| --- |
| **** |
| Scatterplots indicating the range of observed values (x-axis) for each variable against probability of an event (left sided y-axis). Each dot indicates a cardiac catheterization procedure with an event (red) or non-events (green. Blue line indicates a LOESS regression fit divided into low (light blue), medium (mid-blue) and high risk (dark blue) ranges as marked. PR = pulmonary regurgitation; RV = right ventricular; TR = tricuspid regurgitation. |

## Supplementary Statistical Analysis

### Methods

All statistical analysis was performed using R version 4.0.2 (R Foundation for Statistical Computing, Vienna, Austria; [www.r-project.org](http://www.r-project.org)) using RStudio version 1.3.1073 (Boston, Mass). Analysis code is publicly available on Github under a GNU General Public License (<https://github.com/timdawes/paediatricPH>) and summarized in Figure 1. Personal data are not available due to privacy restrictions. Variables are reported as mean ± standard deviation or median (inter-quartile range).

The association of pre-operative factors with adverse events was examined by Bayesian univariate mixed-effects logistic regression (*lme_imp* function, *JointAI* package) with normal priors and appropriate link functions for logistic and continuous outcomes. Missing data are common in observational studies due to differences in reporting frameworks, incomplete or variable documentation. Bayesian joint-modelling allows outcomes to be modelled ’jointly’ with missing data with Markov Chain Monte Carlo sampling from the conditional distributions of the unknown parameters and missing values. This avoids uncongenial models which may occur when these processes are uncoupled. Bayesian approaches allow prior knowledge to be integrated and provide best estimates for the influence of covariates. Supplementary Statistical Analysis includes a sensitivity analysis and a power calculation performed by simulation, based on 80% statistical power, α=0.05, to detect differences in outcome between patients with and without echocardiographic abnormalities using effect sizes published from our institution (1).

Pre-operative continuous variables associated with adverse events at p<0.05 were used in the multivariable model. To develop a multivariable scoring system which was straightforward to use clinically, each variable was divided into ranges representing “low”, “medium” and “high” risk. Category boundaries were optimized by fitting a non-parametric local regression (LOESS) to variable category plotted against event frequency with boundaries between risk categories optimized by k-means clustering (*kmeans* function, *stats* package). This method clusters variable values into ‘k’ (here: 3) predicted risk groups. Once categorized, each variable was added to a multivariable model to predict adverse events using Partial Least Squares regression (*plsRglm* function, *plsRglm* package) on a large number (n=1000) of bootstrapped samples. The median regression coefficients were extracted and rescaled, so that patients with “low”, “medium” or “high” risk characteristics in all variables scored a total of 0, 50 and 100 points respectively. Inference was calculated by permutation testing (2). Missing fields were imputed using a Nonlinear Iterative Partial Least Squares (NIPALS) algorithm (*impute.nipals* function, *mixOmics* package) with five principal components (3).

Model performance was assessed by area under the curve for receiver operating characteristics (AUC_roc_, baseline performance: 0.5) and for precision-recall (AUC_pr_). Precision-recall curves evaluate the proportion of true events within positive predictions and are more informative when positive events are rare. Baseline performance for AUC_pr_ is equal to the fraction of positive observations in the whole cohort (4).

To avoid the over-optimistic estimates of model accuracy which come from developing and validating models on the same data, an ‘optimism bias’ was calculated, and corrected for, using a bootstrap procedure in line with published guidelines and other clinical studies (5-7). This procedure was as follows: the initial prediction model was fitted and tested on the full patient sample (size n), with its apparent accuracy reported using AUC_roc_ and AUC_pr_. The optimism-bias was then calculated by generating a bootstrap sample (also size n) by sampling with replacement from the full patient sample. A prediction model was fitted using this bootstrap sample and tested on the bootstrap sample (bootstrap performance) and on the full training sample (test performance), with accuracy reported by AUC_roc_ and AUC_pr_. The difference in test performance on the bootstrap and full samples was defined as the ‘optimism bias’. Optimism bias was calculated for a large (B=1,000) number of bootstrap samples and the mean optimism bias subtracted from the apparent accuracy to provide an optimism-corrected estimate of AUC_roc_ and AUC_pr_.

$${AUC}_{corrected}={AUC}_{full}^{full}- \frac{1}{B}\sum_{b=1}^{B} ({AUC}_{boot}^{boot}- {AUC}_{boot}^{full})$$

In the equation above, ${AUC}_{x}^{y}$ refers to the area under the curve trained and tested on samples *x* and *y*, respectively, where *boot* is a bootstrap sample of the full sample, and *full* is all the patients in the full sample. The first term ${AUC}_{full}^{full}$ (the apparent model accuracy) is inflated by training and testing on the same sample. This figure is ‘corrected’ by subtracting the second term, the optimism bias for each bootstrap (b) averaged over B samples, providing a more realistic estimate of performance when this model is applied to external data (6, 8-10).

Event probabilities were calculated from the multivariable logistic model, with 95% confidence intervals defined using ±1.96 standard errors of the link values. The derived scoring system was then applied to the original patient group, scores for each patient were calculated (0-100) and models to predict each outcome (escalation of care, complication and no adverse event) were fitted by logistic regression (*plsRglm* function, *plsRglm* package). The association of a patient’s pre-operative score with the occurrence of an adverse event was confirmed by mixed-effects logistic regression (*glmer* function, *lme4* package). Prediction of fatal adverse events was not modelled as events were rare.

### Sensitivity Analysis

A sensitivity analysis to a range of priors was performed to establish model stability in the initial variable selection process, as recommended by recent guidelines. Sensitivity analysis allows the suggested model results to be placed within the context of results that would have been obtained using different priors, thereby providing an estimation of the influence of the chosen priors.

A range of priors was chosen to exceed the expected effects of covariates on the outcome of interest. In this way, the range of results obtained during the sensitivity analysis should exceed the possible range of effect sizes. Prior effect sizes were chosen with narrow ‘informative’ distributions and wide, ‘non-informative’ distributions to investigate whether this also influenced the final model. For the sensitivity analysis, the survival analysis was repeated with prior normal distributions of standardized beta coefficients with all combinations of mean -1, 0, +1 and precisions of 1, 0.1, 0.01, 0.001 and 0.0001 (Supplementary Figure 5). Parameter estimates were similar between choices of prior distributions and importantly no parameter was selected, or de-selected on the basis of choice of prior distribution.

| Supplementary Figure 5 |
| --- |
|  |
| Dot-and-whisker plot of the regression coefficients from univariable Bayesian logistic regression models for adverse events. For each echocardiographic parameter (y-axis) a range of normally distributed coefficient priors were tested, with means (μ) and precisions (τ) as shown in the legend. The strength of association (β coefficients) for all prior distributions are grouped by covariate with mean and 95% credible intervals shown by dots and bars, respectively. A significant association between each parameter and fewer (left) or greater (right) adverse events is shown by deviation of the 95% CI beyond the dotted vertical line at β = 0. Pr(>0) = probability that the effect size is greater than zero. CI = credible interval; LV = left ventricular; PR = pulmonary regurgitation; TR = tricuspid regurgitation; RA = right atrial; RV = right ventricular. |

### Power Calculation

A power calculation was performed by simulation to estimate sample size. Analysis code is publicly available on Github under a GNU General Public License (<https://github.com/timdawes/paediatricPH>/samplesize). Incidence of cardiac arrest (outcome) and echocardiographic abnormality (independent variable) from our institution (1) were used to form a two-by-two contingency table. A Bayesian analysis of these data was conducted by a Poisson sampling scheme. This assumes that the cell counts are Poisson distributed and that the posterior distribution is calculated from the product of the prior and a Bayes factor calculated from the data (*contingencyTableBF* function, *BayesFactor* package). Samples of size n were drawn from the posterior distribution, from which an odds ratio and effect size (beta coefficient). The beta coefficient was defined as the logarithm of the odds ratio. An effect size, and beta coefficient, was calculated for each echocardiographic parameter.

Study groups were then simulated at a variety of sample sizes (Supplementary Figure 6). The distribution of data for each echocardiographic parameter was sampled from a Poisson distribution on a Likert scale (1-7). The Poisson distribution was structured to replicate the incidence of normal echocardiographic parameter seen in previous similar data (1), by setting the Poisson parameter as follows:

$$\lambda= -\ln\left( 1-baseline incidence \right)$$

The outcome was modelled by a binomial distribution (*rbinom* function, *stats* package), with probability of an event set as:

$$p= \frac{e^{L.\beta}}{\left( 1+ e^{L.\beta} \right)}$$

Where *L* = echocardiographic parameter and β = effect size.

Effect sizes varied between echocardiographic parameters resulting in differing estimates of the ideal sample size. A decision was made that at least four echocardiographic parameters should be included in the predictive model, with sample size chosen to anticipate achieving >80% statistical power for ≥4 parameters at α=0.05.

| Supplementary Figure 6 |
| --- |
|  |
| Scatterplot of the results of power calculation by simulation. Study power (y-axis) for association with perioperative cardiac arrest is modelled for sample sizes between 0-300 patients (x-axis). Each dot represents study power when assessed in 500 simulated trials at each sample size, for each echocardiographic parameter. Lines represent a locally-fitted regression fit (solid coloured), 80% power (horizontal dotted) and the number of patients at which four parameters achieve >80% power (vertical dotted). IVS = interventricular septal; RV = right ventricular; TR = tricuspid regurgitation. |

## References

1. Taylor CJ, Derrick G, McEwan A, et al. Risk of cardiac catheterization under anaesthesia in children with pulmonary hypertension. Br J Anaesth. 2007;98(5):657-61.

2. Pesarin F, Salmaso L. The permutation testing approach: a review. Statistica. 2010;70(4):481-509.

3. Wold H. Nonlinear Iterative Partial Least Squares (NIPALS) Modelling: Some Current Developments. In: Krishnaiah PR, editor. Multivariate Analysis–III: Academic Press; 1973. p. 383-407.

4. Saito T, Rehmsmeier M. The precision-recall plot is more informative than the ROC plot when evaluating binary classifiers on imbalanced datasets. PLoS One. 2015;10(3):e0118432.

5. Moons KG, Altman DG, Reitsma JB, et al. Transparent Reporting of a multivariable prediction model for Individual Prognosis or Diagnosis (TRIPOD): explanation and elaboration. Ann Intern Med. 2015;162(1):W1-73.

6. Smith GC, Seaman SR, Wood AM, et al. Correcting for optimistic prediction in small data sets. Am J Epidemiol. 2014;180(3):318-24.

7. Faraoni D, Vo D, Nasr VG, et al. Development and Validation of a Risk Stratification Score for Children With Congenital Heart Disease Undergoing Noncardiac Surgery. Anesth Analg. 2016;123(4):824-30.

8. Efron B. Estimating the error rate of a prediction rule: some improvements on cross-validation. J Am Stat Assoc. 1983;78:316-31.

9. Efron B, Tibshirani R. An Introduction to the Bootstrap. New York: Chapman & Hall; 1993.

10. Harrell FE, Jr., Lee KL, Mark D. Tutorial in biostatistics: multivariable prognostic models: issues in developing models, evaluting assumptions and adequacy, and measuring and reducing errors. Stat Med. 1996;15:361-87.
